# Social Mobility and Political Regimes: Intergenerational Mobility in Hungary, 1949–2017

**DOI:** 10.1007/s00148-021-00875-w

**Published:** 2021-10-08

**Authors:** Paweł Bukowski, Gregory Clark, Attila Gáspár, Rita Pető

**Affiliations:** 1 Centre for Economic Performance, London School of Economics and Political Science (LSE), London, UK; 2 Institute of Economics, Polish Academy of Sciences, Warsaw, Poland; 3 Department of Economics, University of California, Davis, CA 95616 USA; 4 Department of Economic History, LSE, London, UK; 5 Department of Economics and Management, University of Padua, Padua, Italy; 6 Institute of Economics (KRTK KTI), Centre for Economic and Regional Studies, Budapest, Hungary

**Keywords:** Social mobility, Status inheritance, Institutions, Transition, J62, N34, P36

## Abstract

This paper measures social mobility rates in Hungary during the period 1949 to 2017, using surnames to measure social status. In those years, there were two very different social regimes. The first was the Hungarian People’s Republic (1949–1989), which was a communist regime with an avowed aim of favouring the working class. The second is the modern liberal democracy (1989–2017), which is a free-market economy. We find five surprising things. First, social mobility rates were low for both upper- and lower-class families during 1949–2017, with an underlying intergenerational status correlation of 0.6–0.8. Second, social mobility rates under communism were the same as in the subsequent capitalist regime. Third, the Romani minority throughout both periods showed even lower social mobility rates. Fourth, the descendants of the eighteenth-century noble class in Hungary were still significantly privileged in 1949 and later. And fifth, although social mobility rates did not change measurably during the transition, the composition of the political elite changed rapidly and sharply.

## Introduction

Concerns about free-market capitalism in recent years include limited economic opportunity for the lower class and low rates of intergenerational social mobility (Aaronson and Mazumder 2008; Lee and Solon 2009; Olivetti and Paserman 2015; OECD 2018; Elliot Major and Machin 2018; Piketty 2020). Calls for institutional change have intensified during the COVID-19 pandemic, which has disproportionately affected less-affluent people and likely impaired the future prosperity of their children (Elliot Major and Machin 2020; Blundell et al. 2020; Adams-Prassl et al. 2020). However, evidence that social and economic institutions significantly influence social mobility rates is limited.^1^ Measured social mobility rates differ across countries, but is this a function of differences in social and economic institutions, in population composition or in the many other ways in which countries vary?

In this paper we look at Hungary, where a fairly homogeneous population experienced two very different political, economic and social regimes between 1949 and 2017 — communism (1949–1989) and free-market capitalism (1989–2017) — and measure whether the regimes had any effect on rates of social mobility. We measure the social status of different groups within each regime by looking at the status of classes of surnames. We identify four sets of surnames in Hungary, two of high-status and two of low. The high-status surnames are first those ending in *..y*, which was a traditional upper-class surname type in Hungary as far back as the eighteenth century. These are the names associated with the traditional Hungarian noble classes (though the association is not deterministic). Additionally, we identify any surname that was highly over-represented among high school graduates between 1920 and 1939 compared with its estimated population share. The low-status surnames are the 20 most common surnames in Hungary and the surnames that were under-represented in high school education between 1920 and 1939 relative to their population share.

We then calculate the estimated average status of these surnames during the years 1949–2017 by comparing their representation among various elites (education, general and political) relative to their share in the general population. From this, we obtain decadal estimates of social mobility, with those for 1950–1989 showing mobility during the communist era, and those for 1990–2017 showing mobility during post-communist free market capitalism.

We find that social mobility rates throughout were low for both upper- and lower-class families, with an underlying intergenerational correlation of status in the range of 0.6–0.8.^2^ Second, there was no greater rate of social mobility in the communist era than in the subsequent free-market regime.^3^ Third, surnames associated with the Romani minority throughout this period showed even lower social mobility rates, and indeed we see divergence towards lower social status over time, even during the communist era. Fourth, the descendants of the eighteenth century upper classes in Hungary were still significantly privileged during the period 1949 to 2017. Finally, we find that the political representation of the surname groups changed starkly with regime changes, which makes the apparent lack of effect of the transition to democracy in 1989 more striking.

## Historical context

Hungary suffered a devastating loss in World War II. The Red Army crossed its border in late 1944 and started what would become an almost 47-year-long occupation. The Soviets, as they did in the rest of occupied Central and Eastern Europe, soon installed a communist puppet government. As the Iron Curtain came down, Hungary became a founding member of Comecon and later the Warsaw Pact, the respective economic and defense organizations of the Eastern Bloc.

Countries under Soviet occupation followed a remarkably similar political and economic path over the following decades (Fowkes 1993). In all of them the left-wing parties became united under the leadership of the Stalinist hardliners during the year 1948. Centrist, agrarian and moderate right-wing parties were either abolished (as in Hungary) or were reduced to a satellite status (as in East Germany). Political events followed very similar patterns with show trials of non-communists and communists alike; persecution of any dissent; setting up all-knowing secret police; harsh repression that in almost all countries triggered a revolutionary response from society at one point. The communist parties themselves, despite having rather different organizational and sociological origins (Seton-Watson 1958), evolved quite similarly later on (Hanley 2003).

Communist countries of the Eastern Bloc undertook similar, transformative economic and social policies. Some form of land reform took place everywhere as early as 1945, followed by forced collectivization from around the year 1948, which went on full-swing until the mid 1950s, and was completed by the 1960s. Industry was gradually nationalized as part of a switch from a free-market to a planned economy, starting from the biggest manufacturing firms and banks, then proceeding to the middle-sized enterprises, down to the small family-owned businesses. By 1952, the share of the socialized sector was between 77% and 100% in industrial output. In the trade sector the range was 54% to 98% (Swain and Swain 2017). In both dimensions East Germany represented the least collectivized end of the spectrum, while Bulgaria was the opposite, and Hungary was around the median (exactly the median with 97% rate of industrial collectivization, and close to the median of 88% with its own 82% in terms of trade collectivization). Nationalization of private property (land, real estate, businesses, assets) thus took place everywhere with some local differences; Hungarians were more likely to keep their residential real estate, while agricultural collectivization was much less intense in Poland (Hanley and Treiman 2004).

Besides the fundamental change in the ownership structure of the means of production, all Eastern Bloc countries started forced industrialization, with around 50% rates of investment into industry and around a mere 10% into agriculture (Swain and Swain 2017).

Communist countries reformed education as well. Enrollment in secondary education expanded rapidly everywhere, and became almost universal; the expansion of tertiary education was less steep, but enrollment rates increased and reached double digits. This facilitated access, but parental education’s role in explaining children’s educational attainment even increased over time (Nieuwbeerta and Rijken 1996).

Social mobility studies that looked at occupation category correlations of parents and children under communism found that social mobility rates across Eastern Bloc countries were similar to one another throughout the whole period (Domański 1998; 1999). The wage structure in all communist countries (including Hungary) was compressed; returns to skills were much smaller compared to Western countries or to returns after transition to capitalism later on, which brought a large and rapid increase in income inequality (Matvejuu and Lim 1995; Chase 1998; Brainerd 1998; Kertesi and Köllő 1999; Münich et al. 2005). Milanovic (1999) finds that Gini coefficients of income were rather similar (between 19.8 and 25) before transition in six Eastern European countries (Bulgaria, Hungary, Latvia, Poland, Russia and Slovenia), and increased everywhere later on, with Hungary experiencing less increase than most other countries, but still very close to Poland, Slovenia and Latvia.

Besides the explosion of the rigid wage structure, the other major change of transition was the restitution of confiscated property. Hungary chose voucher compensation (with a cap on value); major industrial companies were sold off for cash rather than returned to their former owners (Kozminski 1997). Hanley and Treiman (2004) find similar rates of property ownership in Hungary compared to other former communist countries in the early 1990s.

## Social mobility and institutional change

The most popular formal economic model of social mobility is Becker and Tomes (1979). The authors argue that social status for any individual has two components: a transitory component, which is not transmitted to subsequent generations, and a persistent component that is strongly transmitted. As explicated by Solon (1999) the model assumes a parent (generation *t* − 1) and one child (generation *t*), where the parent allocates their lifetime earnings *y*_*t*− 1_ between their own consumption *C*_*t*− 1_ and investment *H*_*t*− 1_ in the child’s earnings capacity. Parents cannot borrow on behalf of their children to invest in their human capital because of imperfect capital markets. With this specification:

1 $$ y_{t} = (1+r)H_{t-1}+ E_{t} $$

where *r* is the return to human capital investment, and *E*_*t*_ is child ability. It is also assumed that ability is inherited from the parent, but with random components:

2 $$ E_{t} = e_{t} + u_{t}=\lambda e_{t-1} + v_{t} + u_{t} $$

Suppose that the parent has a Cobb-Douglas utility function in *C*_*t*− 1_ and *y*_*t*_, with weight *α* on their own consumption. Equating the marginal utilities from own consumption with child’s income under the budget constraint yields the following optimal level of investment in child’s human capital:

3 $$ H_{t-1}^{*} =\frac{1-\alpha}{1+\alpha r}y_{t-1}-\frac{\alpha}{1+\alpha r}(\lambda e_{t-1}+ v_{t} + u_{t}) $$

It is clear from this equation that parents with higher income invest more in their child’s human capital. The effect of ability, however, is ambiguous. On the one hand, parents with high ability have higher income and thus can invest more. On the other hand, high ability parents expect that their children will also be of high ability, so current consumption yields relatively higher utility for them. The overall effect of ability is positive when the weight on own consumption and/or the rate of intergenerational transmission of ability are low.^4^

The correlation between parents’ and child’s lifetime income in the steady state is:

4 $$ \rho = \delta \beta + (1-\delta)\frac{\beta+ \lambda}{1+\beta \lambda} $$

where *β* = (1 + *r*)*α*, and $\delta = \frac {\alpha ^{2} {\sigma _{u}^{2}}}{(1 - \beta ^{2}){\sigma _{y}^{2}}}$.

This model has few predictions about the effects of different social and political regimes on social mobility. The communist takeover brought almost a complete elimination of income from private capital and a substantial compression of wages through centralization of wage-setting process (Atkinson and Micklewright 1992). This can be conceptualized in the Becker and Tomes model as a reduction in the return to human capital investment *r*. A falling return reduces the dispersion of income ${\sigma _{y}^{2}}$ (Mavridis and Mosberger 2017), without influencing the dispersion of ability ${\sigma _{u}^{2}}$.

The fall of the rate of return to human capital investment reduces the intergenerational correlation of income through two channels. First, by directly changing the relative prices of consumption and children’s lifetime earnings in the parents’ utility maximization problem. That is, the lower rate of return makes investment in children’s earnings relatively less attractive compared to consumption. Second, indirectly through reduced dispersion of income. Since the investment in children’s human capital increases with parental income, compression of the distribution of income also reduces the dispersion of parental investment.

Conversely, the transition from communism to capitalism signified a substantial rise in the return to human capital investment (Campos and Jolliffe 2003; Keane and Prasad 2002), leading to a rise in the dispersion of income (Mavridis and Mosberger 2017) without altering the dispersion of ability. The growing *r* should thus increase the intergenerational correlation of income directly and through the increase in the dispersion of income.

Many other arguments on the potential negative effect of switching from communism to capitalism on social mobility have been articulated in the voluminous economic and sociology literature on socio-economic inequalities under communism (Bergson 1944; Morrisson 1984; Atkinson and Micklewright 1992; Hanley and Treiman 2004). A large portion of all wealth was nationalized under communism, and in all countries some form of restitution took place after transition to capitalism; under communism, downward job mobility of former elites was enforced in some areas; policies aimed at equalizing opportunities and enhancing mobility were implemented upon communist takeovers, which were later lifted etc.^5^

There are, however, several arguments on why social mobility might not necessarily be different under communism and capitalism. The Becker and Tomes model is a model of the transmission of permanent income across generations, where human capital plays a significant role, but the transmission of human capital itself is not explicitly modeled. Parental investment in the human capital of the child can take the form of a transfer of physical or financial assets, or the investment of parental productive time (which could have been used for generating income). Therefore, parents face a trade-off between their own consumption and the future earnings of their children. In reality, however, parents might influence the latter without sacrificing own consumption, for instance, by choosing residential location, providing access to social networks or sharing books and knowledge (Chetty et al. 2014; Chetty and Hendren 2018a; 2018b; Bell et al. 2019). If this is the case, the differences in the dispersion of income across social regimes might not matter for the intergenerational correlation of status.

The simple version of the model does not consider the existence of capital markets, which weakens the connection between parental income and investment in human capital of the offspring. The introduction of capitalism after 1989 brought a development of the capital market in Hungary. Although low-income individuals were still facing significant credit constraints (Popov 2014), the financial market provided options, which were not available under communism. The positive effect of the broadening access to credit on the intergenerational social mobility could thus partially offset the negative effect of higher income inequality.

Well-known features of socialism, such as shortages, queuing, or preferential access to closed shops or certain services by the *nomenklatura* (Bergson 1984; Atkinson and Micklewright 1992) could imply an existence of an informal cap on consumption. Therefore, high-income parents might invest relatively more in the human capital of children compared to a regime with the same level of income inequality, but no constraints on consumption. Consequently, social mobility rates under communism might be relatively low despite the significant reduction in the dispersion of income. However, the general consensus in the literature is that the non-monetary aspects of consumption and earnings under communism did not systematically favour low- vs. high-income families (Bergson 1944; Morrisson 1984; Atkinson and Micklewright 1992; Milanovic 1998).

Finally, others have argued that we should not necessarily see an abrupt change in social stratification (and as a consequence, mobility) upon transition to capitalism, because status transmission is mostly governed by education in all industrialized countries (communist and capitalist alike); or because the skills (or connections) that determined elite status in communism were readily usable, or convertible to capital under capitalism (Hanley and Treiman 2004).

## Measurement of social mobility

### Empirical model

We follow studies of social mobility rates at the group level (Güell et al. 2007; Collado et al. 2012; Clark and Cummins 2014; Clark et al. 2015). We implement the methodology in Clark and Cummins (2014) and model observed status for any individual as a function of a persistent, group level component, that is strongly transmitted across generations, and a transitory, individual level component, which is not transmitted. Our measure of social mobility is the intergenerational correlation of the group level component of social status. We chose this method because it uses data that is relatively easily accessible, the general surname distribution of the population and name lists of members of the elite groups. Torche and Corvalan (2018) show analytically that total social mobility (i.e. the persistence of an outcome between a pair of an adult child and their parent) is a weighted average of the persistence of the individual level components and the persistence of the group averages, where the weights are given by the respective variance share of the individual and the group level components. Accordingly, our findings could be interpreted as between-group estimates of social mobility.

In this framework, the status *y* of each individual *i* from group *g* in each generation *t* is composed of an underlying group-level component *x* and a transitory component *u*:

5 $$  y_{it}^{g}={x_{t}^{g}}+u_{it}^{g} $$

Group level status is inherited strongly at the rate *ρ* with non-negative multiplicative error *e*_*it*_, so that the latent status of group *g* at time *t* is:

6 $$  {x_{t}^{g}}=\rho x_{t-1}^{g} \cdot e_{it}. $$

In order to estimate the social mobility rate in Hungary (i.e. the intergenerational correlation *ρ*), we need to construct a measure of the latent mean social status ${x_{t}^{g}}$. In what follows, we present a methodology of estimating ${x_{t}^{g}}$ using the data on membership in various elites: education, general and political. The educational elites are graduates from medical and technical universities. The general elites are captured by patenting inventors and people listed in “Who is Who” books. The political elites consist of members of the Hungarian Parliament and members of the Hungarian Academy of Sciences.

We define social groups *g* as groups of individuals with surnames of particular origin. In particular, we identify traditional upper- and underclass surnames in Hungary (we discuss this in detail in the next subsection) and we treat all individuals with such surnames as members of either upper or lower class.

The idea is to infer the latent mean social status of certain surname groups from their membership in elites. This approach requires two types of data. The first is the population shares in Hungary of traditional upper- and underclass surnames. The second is the shares of these surnames in various elites. In addition, we must make the following three assumptions:

- Social status in Hungary is normally distributed with constant variance across generations ($u_{it}^{g}\sim N(0,{\sigma _{g}^{2}})$).
- The target surname groups had the same variance of social status as the population as a whole among their members (*σ*_*g*_ = *σ* for all *g*).
- Members of the elite represent some portion (*α*_*t*_*%*) of the top of the social status distribution in Hungary.

We specify *α*_*t*_*%* in a way that it tracks potential changes in the relative “eliteness” of the occupation (the exact method is somewhat different across elite groups, so we discuss it in detail in Section 4.3 below). In the Appendix of Bukowski et al. (2021) we show that the results are virtually unchanged if we assume that *α*_*t*_*%* = 1*%* across all elites, which is the approach taken by Clark (2015).^6^

Suppose an individual enters the elite if her status is above a time-variant threshold (which is common across groups):

$$ y^{g}_{it}>\underline{y}_{t}. $$

The probability that a current member from group *g* enters the elite is:

$$ P_{elite}^{g}=P({x_{t}^{g}}+u_{it}^{g}>\underline{y}_{t})=1-P(u_{it}^{g}<\underline{y}_{t}-{x_{t}^{g}})=1-{\varPhi}\left( \frac{\underline{y}_{t}-{x_{t}^{g}}}{\sigma_{g}}\right), $$

where *Φ* is the cumulative distribution function of the standard normal distribution. We can express the same equation in the following way:

7 $$ \frac{\underline{y}_{t}-{x_{t}^{g}}}{\sigma_{g}}={\varPhi}^{-1}\left( 1-P_{elite}^{g}\right) $$

This relationship holds also for the entire population. Without a loss of generality, assuming that *x*_*t*_ = 0 (which means that ${x_{t}^{g}}$ is defined relative to the social mean) the equation in this case becomes:

8 $$ \frac{\underline{y}_{t}}{\sigma}={\varPhi}^{-1}\left( 1-P_{elite}\right), $$

where *P*_*elite*_ is the overall exclusiveness of the elite. This shows that the threshold for entering the elite is implicitly pinned down by *P*_*elite*_ and *σ*. Subtracting 7 from 8:

9 $$  {\varPhi}^{-1}\left( 1-P_{elite}\right)-{\varPhi}^{-1}\left( 1-P_{elite}^{g}\right)=\frac{{x_{t}^{g}}}{\sigma_{g}}-\underline{y}_{t}\left( \frac{1}{\sigma_{g}}-\frac{1}{\sigma}\right) $$

The first term on the left hand side is the same as *α*_*t*_*%* from assumption (c). How we calculate exclusivity differs across data sources, so we deal with this in Section 4.3.

The second term we can calculate from the relative representation of group *g* in the elite. The relative representation is the ratio of the group’s share in the elite and its population share.^7^

Given assumption (b), the second term of the right hand side of Equation 9 disappears (i.e. the estimated social status does not depend on the threshold $\underline {y}_{t}$). In Appendix A (page 56) of Bukowski et al. (2021), we illustrate the potential bias resulting from the violation of this assumption, but we also show that assuming different variances has a relatively small effect on the estimates empirically, and that it converges to zero over time.^8^

**Fig. 1:**
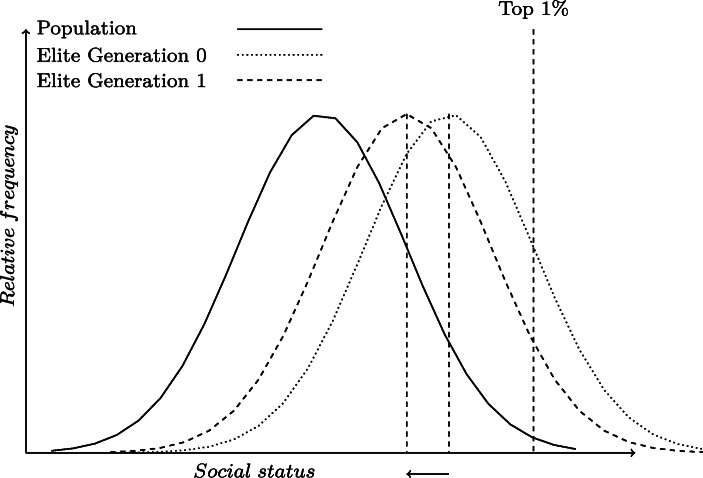
Illustration of estimating social mobility rates from surname distributions. The figure shows how we infer latent social status *x*_*t*_ of a certain group from its observed shares in the elite. The solid line represents the status distribution of the population. We assume that 1% of the whole population makes it into the elite, which defines the cutoff level of social status needed to join the elite (represented by the Top 1% vertical dashed line). Next we turn to the dotted curve, which is the status distribution of the group in the 0^*th*^ generation. Knowing the share of the group who made it to the elite (the mass of individuals beyond the cutoff of Top 1%) and assuming that the variance of the group’s distribution is the same as the population, we can infer its mean relative to the population mean. Doing the same with the next generation data, we can infer the speed of convergence to the mean over a generation for the group

Figure 1 illustrates the intuition on how we attribute to each surname group in each period an implied average social status. If we know how over or under-represented a group is among the elite we can then estimate its mean social status. Assuming medical graduates, for example, represent the top 1% of the distribution, if we observe that a particular surname type has 3% of its members found among medical graduates, then this will translate into that group having an average status that is 0.45 standard deviations above the social mean.

Having estimated the implied mean of status for an upper- or underclass surname type in each decade during 1949–2017, we can then calculate for each decade the implied correlation of status *b*10*g* with the previous decade. From 5 and 6:

10 $$ \ln{x}^{g}_{t}=\ln{x}^{g}_{0}+\ln b^{g}_{10}\times t+{\ln\epsilon^{g}_{t}} $$

where *𝜖tg* is an error term corresponding to mis-measurements, and *b*10*g* is the correlation of status across a decade. We can estimate $\ln b^{g}_{10}$ by regressing the logarithm of the calculated latent status on a constant and a time trend using OLS. Then, assuming that a generation is 30 years, the implied intergenerational correlation of underlying status for group *g* is given by

11 $$ \rho^{g}=(b^{g}_{10})^{3} $$

### High and low-status surnames

The first high-status surname group we focus on are surnames ending in *..y*, which in our study period constituted typically 2% of the population.^9^ In pre-modern Hungary, there was a set of surnames that could be written with either an *..i* or *..y* ending. These surnames supposedly signified a location from which the family is originally from, or where their family estates were located. The *..y* spelling was considered more archaic and elegant very early on, and became widely associated with the nobility.

Perhaps the most famous example for this class of surnames is former French president Nicolas Sarkozy, whose father (Pál István Ernő Sárközy de Nagy-Bócsa) was born to a family of the Hungarian lesser nobility that gained its title in the seventeenth century fighting the Ottoman Empire. 29% of all Hungarian prime ministers since 1848 came from a *..y* named family, which corresponds to an average rate of over-representation of a factor of 15 compared to the current population share of such names. We see large variation across political regimes (9 out of 21 PMs of Austria-Hungary, 4 of 14 PMs of the interwar far-right regime, exactly 0 communist PMs and 1 out of 7 PMs elected after 1990 belonged to the *..y* named group). The over-representation of *..y* names is not limited to politics, as two of the ten Hungarian Nobel-laureates had a *..y* ending family name (György Békésy and György Hevesy).

Although there is no deterministic relationship between being a noble and having a *..y* ending family name, we are able to demonstrate the elite status of these names as far back as the eighteenth century in a number of ways. In the 1720 census of the taxpayer population (which excluded high nobility), a member of the petty nobility was three times more likely to have a *..y* ending name than non-nobles (14% vs. 5%). In the conscription of the nobility of 1755, which was a list of tax-exempt nobles who were not part of the high aristocracy, the *..y* name share was even higher, at 25%. Finally in the complete list of the land-owning aristocracy in 1767, the *..y* ending covered a full 40% of the high aristocracy. Thus in eighteenth-century Hungary the higher was the status of a given subset of society, the greater was the over-representation of *..y* surnames in it.^10^

At the dawn of the revolution of 1848, some members of the progressive elite with noble backgrounds voluntarily and demonstratively changed their names to the more plebeian *..i* ending. Nevertheless, having a *..y* ending name was closely correlated with military rank even in the revolutionary army (“Honvédség”). In 1848 non-commissioned officers were twice as likely to have *..y* ending name than privates, while commissioned officers were five times more likely (Mikár 1891).

Surnames ending in *..y* were still considered a mark of privilege in the late nineteenth century, and were put under protection when many thousands “Hungarianized” family names which suggested foreign origin.^11^ Consequently, it has been legally impossible to adopt such a name since the 1880s (Karády and Kozma 2002, p.61). In the few cases that a *..y* name was adopted, it was mostly because the family was ennobled at the same time.^12^ Notably, names of archaic orthography, such as those ending in *..y*, are still legally protected in Hungary. The 2010 Law on Civil Procedure states that “historic” (article 4/B of §49) and “archaic” (article 4/C of §49) names cannot be adopted. Thus the majority of holders of *..y* surnames 1945 and later were the descendants of the upper classes of the nineteenth century.

The second elite group is defined based on over-representation in secondary education between 1920 and 1939. We divided the relative frequency of each name among high school graduates by its population share. Then we tagged the names in the upper quartile of the resulting distribution as elite. We also counted as elite those names that appeared among high school graduates, but were too rare to appear in our sample of the general surname distribution.^13^ High school graduates during the period 1920 to 1939 were still only an estimated 2–5% of each cohort, depending on the year.

The first underclass group consists of those with the 20 most common surnames in Hungary in the twentieth century.^14^ These surnames, which are held by 20–25% of the population from 1945 onward, were under-represented among Hungarian educational and occupational elites, including high school graduates, in all periods before 1945. To see why this is the case, we need to look at the history of surname use.

Hungarian society adopted surnames during the high to late middle ages; the nobles were the first to do so, town-dwelling commoners the next, and serfs the last (Karády and Kozma 2002). As keeping track of the lineage was of vital importance to the land holding class, they chose distinctive surnames based on the area they owned (which is the origin of the *..y* ending names), or the name of an ancestor (which is the origin of the ..fi ending names, the Hungarian equivalent of the *..son*/*..sen* ending in Germanic languages). Distinction was less important for commoners, so their family names started out simply as nicknames, which bore reference to the owners’ profession, social status, ethnic origin, or physical appearance. In our group of the most common Hungarian surnames we find 8 (or 9) surnames indicating professions, 6 (or 7) surnames referring to physical characteristics, 5 surnames referring to ethnicity or country of origin (or likeness of such), and one referring to social status.^15^ Surnames (especially those of the common people) at first were not inherited, just used for distinguishing between two people having the same first name; having an inherited, patrilineal surname only became commonplace by the start of the seventeenth century. However, feudalism in Hungary persisted well into the middle of the nineteenth century, so the status of the holders of these names could only have started to regress to the mean three generations prior to our analysis.

The second low-status surname set consists of the surnames that occur at least twenty times more frequently among marriages than among high school graduates during 1920–1939.

The third underclass group is a set of surnames associated with the Romani minority. These were identified first as names that the Hungarian Encyclopedia of Surnames recognizes as Romani surnames. Most of the Romani, however, have common Magyar surnames, so the names we found in this way represent a very small percentage of the population (less than 0.1%). The Romani minority is associated with much higher fertility than the rest of the Hungarian population in recent decades (Pénzes et al. 2018). Thus, we identified also as Romani-associated surnames those with a growth rate of more than 10% between their respective population counts in 1998 and in 2016.^16^

### Data

The estimation of surname-based social mobility measures requires two types of data. The first is the population shares of traditional upper- and underclass surnames. The second is the shares of these surnames in various elites.

#### Population shares

We estimate population shares of surname groups from a sample of marriage records from 1940 to 1951 and the complete population registers of 1998 and 2016, interpolating for the years between 1951 and 1998, and between 1998 and 2016.

The sample of marriages contains 842,000 people, and it was digitalized by the Hungarian Society for Family History Research.^17^ As the goal of the compilers is to digitalize all available records, we assume that the data represent a random sample of all marriages in this period. Before World War II, the average annual number of marriages was 16,672, but after the number dropped to 6,774 marriages annually. The coverage rate as a share of all marriages is 9.5% in 1938, and 2.5% in 1949 (Balázs 1993).

We obtained the complete surname distribution of Hungary in 1998 and 2016 from the Ministry of the Interior. The data includes the list of all surnames and the exact number of people having them, excluding (for privacy reasons) surnames held by a single person.

#### Elite groups

##### Educational elites.

We consider three different sets of elite groups: educational, general and political. We capture educational elites from three data sources. The first and most comprehensive is the distribution of surnames of Hungarian medical school graduates. We have records of all medical graduates from Hungarian universities from 1940 to 2017.^18^ The list of graduates was provided by the State Healthcare Service Center. In order to measure the change in the relative “eliteness” of the medical profession (*α*_*t*_*%*) we calculate the share of all medical graduates as a percentage of the cohort of the 25 years old in every year. The latter information is available at the web page of the Hungarian Central Statistical Office (KSH).^19^ During the whole study period the share of medical graduates remains remarkably stable at around 1% of the respective cohort. Quantitative evidence shows that the medical profession still attracted the best students in the period of 2008 to 2015 (Fábri 2016).

The next educational elite are the PhD graduates of Budapest University of Technology and Economics, whose names we collected from the Millennium Yearbook issued by the university in 2000 (Kiss 2000). It allows us to estimate social mobility rates from 1960 to 2000. In the case of PhD graduates we keep track of the relative “eliteness” by assuming that the group represented the top 1% in the 1960s, and then its exclusivity changed proportionally to the total number of PhD graduates (i.e. during the seventies the number of graduates increased by 46% relative to the sixties, so we assumed an *α*_*t*_ of 1.46%).

Finally, we constructed the list of those who earned a (non-doctoral) university diploma using the university yearbooks that were published on the university website from 1962 to 1999.^20^ Non-medical degree programmes at universities were uniformly 5 years long in the period covered by our data. Because of this, we will refer this group as “masters” (as they earned the equivalent of a combination of a Bachelor of Sciences and a Master of Sciences degree). In their case the relative “eliteness” measure is the share of people with any university degree in the young adult cohort. This way we account for a general university diploma inflation that took place over time.

##### General elites.

We capture general elites by looking at inventors and people mentioned in the Hungarian edition of “Who is Who”. The data on Hungarian inventors come from the worldwide patent statistical database PATSTAT. We create a list of unique inventor-decade-application observations starting from 1970, the year when Hungary joined the World Intellectual Property Organisation. We look at applications instead of granted patents; we do not distinguish between Hungarian and international applications.^21^

The second general elite name set is based on the scanned version of the Hungarian edition of Hübners Who is Who, a collection of biographies of famous people (Gábor et al. 2011).^22^ The Who is Who reflects a general idea of “being famous” for any reason. Unfortunately, we do not know which year a person entered Who is Who, just their year of birth. Because of this, we created a panel of synthetic cohorts where every individual is assigned to the cohort when they turned 23.

In case of the general elites we assumed that in the first decade their relative exclusivity was *α*_1_ = 1*%*, and then adjusted it according to the number of inventors and Who is Who items per decade. So 7265 individuals applied for patents in our sample in the 1970s, and we assign 1% eliteness to this value; if this number increased to 14530 in a subsequent decade, we would adjust the exclusiveness of the inventor group to 2%.

##### Political elites.

Finally, we also look at political elites. We include in this group two sets of names, first is the Members of Parliament, the second is the members of the Hungarian Academy of Sciences.

The first democratic elections after World War II were held in November 1945; parties were free to participate except for prominent parties of the preceding right-wing regime. The subsequent 1947 elections were marked by voter fraud by the Hungarian Communist Party, who won the plurality of the votes, but were still very far from commanding a majority in the National Assembly. They merged with the Social Democrats and took power nevertheless, and between 1949 and 1980 parliamentary “elections” featured a single candidate of what was by then called the Hungarian Working People’s Party (later the Hungarian Socialist Workers’ Party) in each electoral district. The first multi-candidate election took place in 1985, but still the overwhelming majority of candidates were communist party members. After the transition to democracy, the first free and fair election took place in 1990.

We manually collected the list of all members of the Hungarian Parliament since it first convened as an elected, representative legislature in 1848. For the pre-1990 cycles we used three main sources. The primary sources were the Almanacs of the Hungarian National Assembly and the address books of the Hungarian National Assembly. For electoral cycles where these did not provide name lists of the representatives, we used the verbatim records of the first session following the election where the credentials were passed to all newly elected members.^23^ The data source for the post-communist period is the current home page of the Hungarian National Elections.^24^

We complement the picture of political elites with the data on the members of the Hungarian Academy of Sciences.^25^ The Hungarian Academy of Sciences was established from private donations in 1825 as a body of scholars deemed best at their fields whose goal was to preserve and promote Hungarian culture and science. In its present form, new members are elected by current members, and the maximum number of members under the age of 70 is fixed by law at 200 (Act XL of 1994 on the Hungarian Academy of Sciences). Although this recruitment procedure lends a great degree of formal independence to the body, because of the high standing and authority of the members and the body as a whole, the Hungarian Academy of Sciences has always had political importance. Communists in 1949 purged members who were deemed ideologically unfit, whose membership was restored after the democratic transition.

Before turning to the analysis we make three further adjustments. First, to have an overall picture of Hungarian society we exclude foreigners whenever their presence in the data would be an issue. In the case of the medical graduates the State Healthcare Center data lets us directly exclude foreign medical students. In the case of the graduates of Budapest University of Technology and Economics, if the nationalities of the students were listed we used this information to detect foreigners, otherwise, we detected foreign students based on their names. We do not face this problem neither with the general elite data, nor with the political elite data.^26^

Second, as all our sample included women as well, we have to overcome the issue of changing surnames upon marriage. In Hungary, the most common way of changing surname upon marriage is to chose the surname of the husband and augment it with a special ending (“*-né*”) and either keeping the maiden name as a second surname or drop the maiden name entirely. Due to this rule, we can tag married women based on their name, and for most of them, we can recover their maiden name as well. We used the maiden name in the analysis whenever it was possible. We handled this issue in the same way in all the data sources. As a robustness check, we carried out the analysis separately by genders using the medical data (where this information was given), and we found no significant differences in social mobility rates.

Third, the political elites contain very limited number of individuals, even compared to the other elite groups. Elections take place only every 4 to 5 years, and there is a large continuity in membership from one cycle to the next. The composition of the Academy changes even more slowly (most of the time). Also, we cannot make the assumption that people become members of these bodies at a certain age. Consequently, we can only work with relative representation ratios with these data, as our model of latent social status is not applicable in their case. For the Parliament we calculate relative representation ratios over time for each election cycle. For the Academy of Sciences we create a yearly pseudo-panel where the observations reflect the name structure in any given year, and we calculate relative representation figures from this data.

### Descriptive statistics

Table 1 shows the observation counts from each set of elite names. Table 2, Panel A shows the estimated population share of each surname group between 1940 and 2017. We see significant differences between the 1940s and the 1950s due to World War II and its aftermath, which dramatically reshaped Hungarian society and its surname distribution.^27^ Two important features of the data are the gradual decrease in the share of the *..y* surnames (by about 25%) over two generations and the more than two-fold increase of the share of the Romani-associated surnames over the same period. As we show in Section 5.2, the estimates of the actual Romani population share (which are scarce) show a similar trend. Otherwise, the name distribution is very similar in the 1950s as in the 1990s and 2010s.

**Table 1:** Number of observations

Decade	Medical doctors	Technical PhD	Technical master	Inventors	Who is Who	Parliament	Members of the HAS
1950	10115	25				636	49
1960	13313	1198	16174		3524	689	61
1970	10950	1747	28192	7265	11692	704	123
1980	10604	2319	19836	24223	18179	738	88
1990	9745	1750	15294	12522	13624	1212	157
2000	10770			8683	11221	818	117
2010	12663			6836		829	117
Total	78160	7039	79496	59529	58240	5626	712

*Notes*: The table shows the number of people in all elite occupations available to our analysis aggregated to decades. Medical doctors correspond to the sum of Hungarian nationals who graduate from one of the four Hungarian medical faculties (Semmelweis in Budapest, and the universities in the towns of Debrecen, Pécs and Szeged). Technical PhDs and Technical masters correspond to graduates of Budapest University of Technology. Inventors are collected from the PATSTAT database. Who is Who corresponds to names in Hübners Who is Who (Gábor et al. 2011). Members of Parliament are counted in election years and include everyone who wins a parliamentary seat during the election cycle (special elections included after 1990). Members of the Hungarian Academy of Sciences (HAS) in this table are newly elected full members or corresponding members in each decade

**Table 2:** Social status of surname types, 1940–2017 — medical graduates

Decade	*..y* surnames	High-status	20 most	Low-status	Romani-
		surnames 1920–39	common surnames	surnames 1920–39	associated surnames
*Panel A: Population shares*					
1940–49	0.025	0.005	0.232	0.074	0.012
1950–59	0.024	0.005	0.255	0.071	0.013
1960–69	0.022	0.005	0.249	0.070	0.016
1970–79	0.021	0.005	0.243	0.070	0.020
1980–89	0.020	0.006	0.237	0.069	0.023
1990–99	0.019	0.006	0.231	0.068	0.026
2000–09	0.018	0.006	0.229	0.068	0.029
2010–19	0.018	0.006	0.229	0.068	0.032
*Panel B: Relative representation among doctors, vs total population*					
1940–49	4.57	5.80	0.52	0.05	0.81
1950–59	4.01	2.93	0.57	0.67	0.56
1960–69	3.72	3.34	0.66	0.68	0.49
1970–79	3.22	2.57	0.70	0.60	0.43
1980–89	2.50	2.11	0.77	0.62	0.36
1990–99	2.86	2.16	0.80	0.64	0.33
2000–09	2.69	2.05	0.86	0.80	0.31
2010–19	2.64	2.08	0.91	0.79	0.33
*Panel C: Implied mean social status*					
1940–49	0.57	0.68	-0.20	-0.23	-0.07
1950–59	0.51	0.40	-0.18	-0.14	-0.18
1960–69	0.49	0.44	-0.14	-0.12	-0.23
1970–79	0.43	0.34	-0.11	-0.17	-0.28
1980–89	0.34	0.27	-0.07	-0.15	-0.33
1990–99	0.38	0.28	-0.07	-0.14	-0.36
2000–09	0.36	0.25	-0.05	-0.07	-0.37
2010–19	0.36	0.27	-0.02	-0.07	-0.36

*Notes*: Panel A shows the population shares of the groups defined by surname type (see the text for definitions). Panel B shows the relative representation of the surname groups among graduates of medical universities in Hungary. The relative representation is defined as the ratio of the share among graduates to the population share. Panel C shows estimates of mean status expressed as standard deviation units difference above or below the social mean. The mean status is estimated from relative representations (see the text for more details). Appendix Table A4 on page 75 of Bukowski et al. (2021) shows the same measurements using the non-Romani population

## Social mobility, 1949–2017

### Educational elites

Our first set of results concerns the estimates of social mobility using data on medical school graduates. For reasons explained below, we estimate the status of the two high-status social groups (the *..y* ending surnames and the interwar high-status group) and the two low-status social groups (the top 20 most frequent surnames and the interwar low-status group) relative to the non-Romani population. In Table A3 on page 74 of Bukowski et al. (2021) we show the re-calculated population shares and the estimated share of the Romani population over time.

**Fig. 2:**
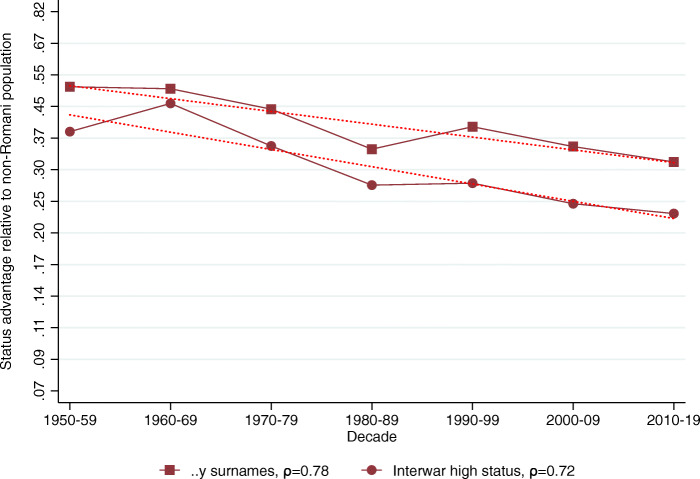
Status persistence of advantaged surnames among medical doctors. The figure plots the implied mean status advantage of the high-status names (in standard deviation units) presented in Table 2, columns (1) and (2) and the linear fit. The status advantage is shown on a logarithmic scale on the vertical axis. The legends contain the calculated *ρ* intergenerational correlation coefficients of status by surname group. A lower correlation means less status persistence, which means more social mobility (steeper line in absolute value)

**Fig. 3:**
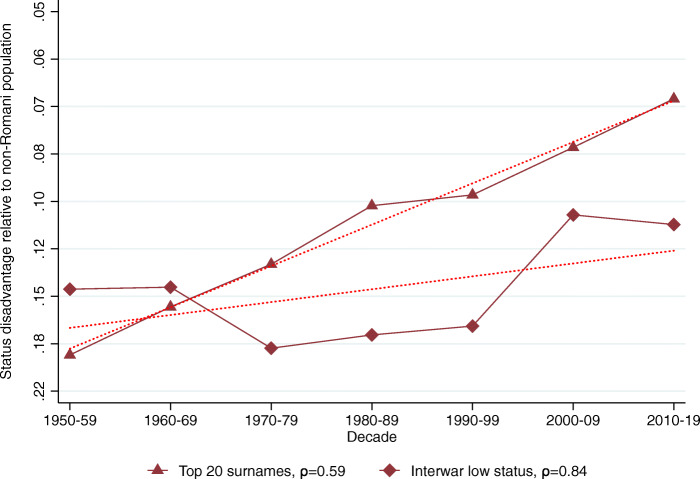
Status persistence of disadvantaged surnames among medical doctors. The figure plots the implied mean status disadvantage of the low-status names (in standard deviation units) presented in Table 2, Columns (3) and (4) and the linear fit. The status disadvantage is shown on a logarithmic scale on the vertical axis. The legends contain the calculated *ρ* intergenerational correlation coefficients of status by surname group. A lower correlation means less status persistence, which means more social mobility (steeper line in absolute value)

**Fig. 4:**
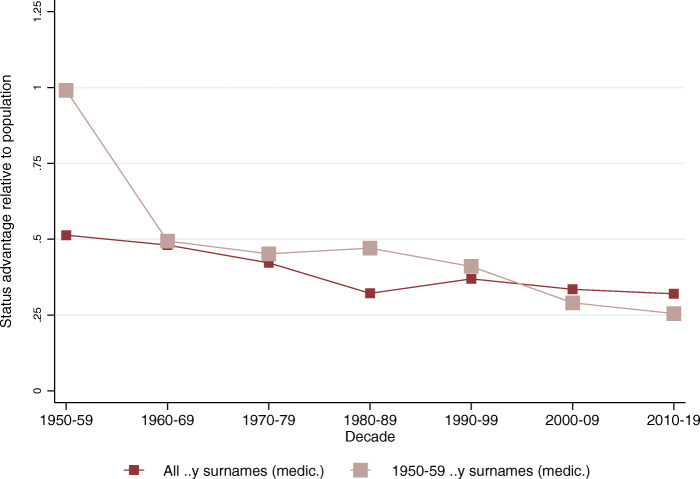
Inheritance of medical status among the *..y* surname group. The figure plots the implied mean status advantage of the *..y* surname group (dark) and the subset of *..y* names who graduated as medical doctors in the 1950s (light). The vertical axis represents standard deviation units of social status

**Fig. 5:**
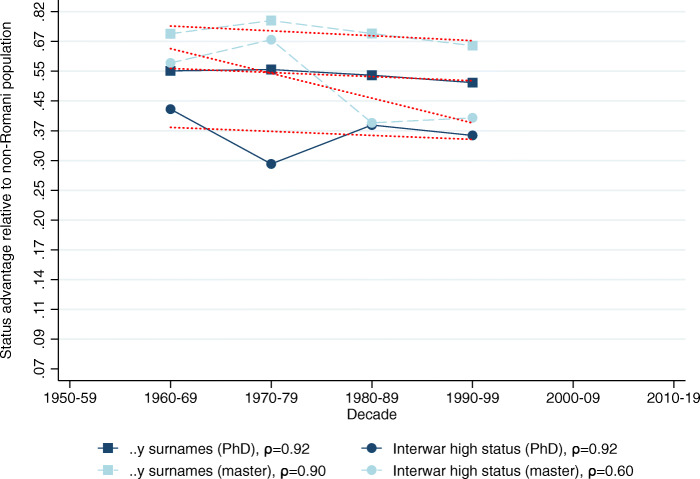
Status persistence of advantaged surnames among technical university graduates. The figure plots the implied mean status advantage of the high-status names (in standard deviation units) and a linear fit. The data points can be found in columns (1) and (2) of Tables A5 and A6 in Bukowski et al. (2021, pages 76–77). The status advantage is shown on a logarithmic scale on the vertical axis. The legends contain the calculated *ρ* intergenerational correlation coefficients of status by surname group. A lower correlation means less status persistence, which means more social mobility (steeper line in absolute value)

**Fig. 6:**
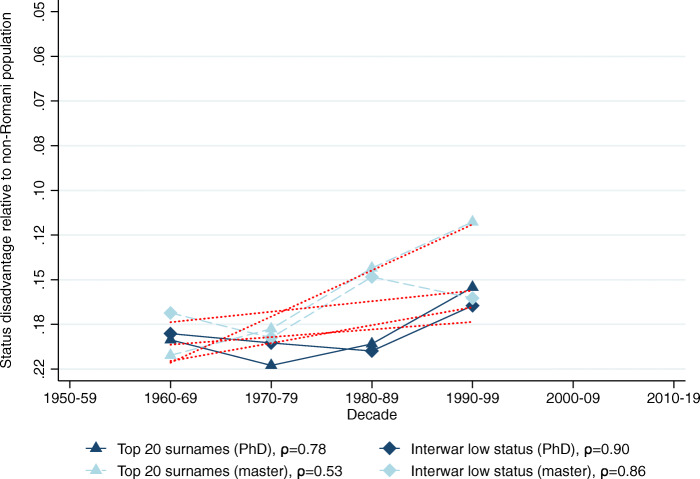
Status persistence of disadvantaged surnames among technical university graduates. The figure plots the implied mean status disadvantage of low-status names (in standard deviation units) and a linear fit. The data points can be found in columns (1) and (2) of Tables A5 and A6 in Bukowski et al. (2021, pages 76–77). The status advantage is shown on a logarithmic scale on the vertical axis. The legends contain the calculated *ρ* intergenerational correlation coefficients of status by surname group. A lower correlation means less status persistence, which means more social mobility (steeper line in absolute value)

The relative representation of the five surname groups among Hungarian medical graduates in 1940–2017 is shown in Table 2, Panel B. Using these data we calculate the implied mean status for each surname group in each decade shown in Table 2, Panel C.^28^ Figures 2, 3, 4, 5 and 6 show the implied mean status by decade compared to the non-Romani population and the implied intergenerational correlation of educational status, assuming a generation is 30 years.

Figure 2 shows the status advantage of high-status names over the study period. We document an intergenerational correlation of *ρ* = 0.78 for the *..y* ending surnames, and *ρ* = 0.72 for the interwar high-status group. The figure shows that at the onset of the communist period the average *..y* ending named individual was about 50% of a standard deviation above the average person in society, and this advantage has only diminished to about 30% of a standard deviation by present times, showing considerable persistence over two generations. The interwar high-status group has a lower status advantage to begin with, but progresses towards the mean by an almost identical (slow) pace. There is also no visible deviation from previous trends after transitioning to capitalism.

Figure 3 shows the status advantage estimates for low-status surnames among medical doctors. The group of the top 20 most frequent surnames progresses to the mean more rapidly, reducing its disadvantage from 18% of a standard deviation below the mean in the 1950s to 7% below the mean in the 2010s. The intergenerational correlation coefficient in their case is *ρ* = 0.59. The interwar low-status group shows a more persistent social status with a correlation coefficient of *ρ* = 0.84.

Though the share of medical graduates relative to the relevant cohort of society is remarkably stable over time, we reproduced the main results imposing the 1% eliteness assumption for the sake of comparability to the other similar results (Figures A10 and A11 on pages 70–71 in Bukowski et al. 2021, the working paper version of this article).

The richness of the medical doctor data allows us to carry out several robustness checks. One alternative interpretation of our results could be that what we measure is not general social status, but the fact that children of doctors are more likely to be doctors themselves. We argue that this is not the case; rather, that high-status persons in a society will be more likely to transfer their social status to their children who will be more likely to take up high-status professions, such as that of a medical doctor (or an engineer, an inventor, or a politician, as we will see). If our results were driven by only within-family transmission of occupation, then if we picked a set of surnames that were over-represented among medical doctors in decade *t*, we would not expect the same names to be over-represented again until decade *t + 3*, as their children would graduate approximately thirty years later. So we would see very low persistence of social status from one decade to the next.

We test this idea formally. In Fig. 4 the dark line presents the implied social mean status of the *..y* ending surnames among medical school graduates (the same as in Fig. 2). The light line represents a new set of surnames: the surnames of those within the *..y* name group who graduated in the 1950s. If within-family transmission drove the results, we would not see any above-mean social status for these names in the 1960s and 1970s when their own children would have arguably not been going to medical school yet. The first feature to note in the graph is that the status of the light-colored social group is very high in the 1950s, which is purely by construction. The estimated social status is based on the measure of relative representation, which is the ratio of the surname’s share among the elite and the surname’s share in the population. For the 1950s *..y* named doctors the numerator is exactly the same as for the general *..y* named group, while the denominator is a much smaller number. The second thing to note is that there is indeed a small bump in the social status of the 1950s doctors’ names in the 1980s, meaning that the occupation probably does transfer to an extent within the family. Most importantly, however, the social status of the 1950s doctor names is virtually identical to the general *..y* named group in every other decade as well, even when this cannot be the result of a direct parent-to-child transfer of occupation. This suggests that the direct within-family transmission of occupation is an unlikely explanation of the overall strong persistence of the *..y* ending surnames.

In the Appendix of Bukowski et al. (2021, Figure A2 on page 63) we also divided the results based on university rank, treating Budapest- and non-Budapest-based medical faculties separately. Semmelweis University, the Budapest-based medical faculty is the oldest and most prestigious medical faculty in Hungary and outranks the non-Budapest medical faculties. While the estimated status persistence rates are remarkably similar, the results confirm the implication of the model that high-status groups should be more over-represented the closer one gets to the top of society. In line with this, we find that the *..y* named surname group’s over-representation is higher in Budapest as it is in the rest of the faculties, while the top 20 most frequent surnames’ under-representation is smaller in the non-Budapest-based faculties. Neither the top 20 nor the *..y* surnames have a geographic distribution within the country that explains this pattern, and all faculties are recruiting from all locations, and usually, the Budapest-based faculty is the first choice for those aspiring for a medical career (Fábri 2016). In Figure A3 of the Appendix in Bukowski et al. (2021, page 64) we also carried out the analysis by gender and found that the results were remarkably similar with somewhat less persistence among females, which is explained by the fact that family name is inherited through the patriline and we have more measurement error with women (some were already married by the time of graduation).

We next consider graduates from the PhD and master’s programmes of the Budapest University of Technology and Economics — the largest and most prestigious technical university in Hungary. Figures 5 (high-status names) and 6 (low-status names) plot the results; we relegated the corresponding data points to the Appendix in Bukowski et al. (2021, Tables A5 and A6 on pages 76–77). The figures show very similar results to the medical doctors. There is a high level of persistence of the high-status names, even higher than before (.90 or more in 3 out of 4 cases). The *..y* surnames are progressing towards the mean more slowly. Similarly, the top 20 names progress towards the mean at a faster pace than the interwar low-status names as they do with medical doctors, and with both low-status surname groups social mobility is slower compared to doctors.

Because we estimated the change in the eliteness of technical PhDs and masters from the data in a different way, the levels are no longer comparable across elite groups, but across social groups within the same elite. In the Appendix of Bukowski et al. (2021, Figures A12 and A13 on pages 71–72) we showed the results where we calculated status advantage and disadvantage levels imposing the constant 1% eliteness hypothesis. These results show that PhDs had higher average status than masters, meaning that high-status names had a larger advantage in PhDs as they did in masters, while low-status names had a worse disadvantage. However, these results overestimate *ρ* and misinterpret degree inflation as an increase in social mobility.

### The non-convergence of the Romani

**Fig. 7:**
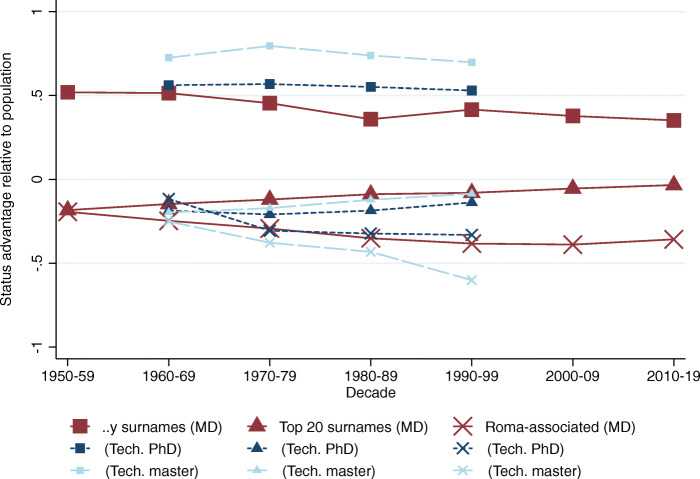
Mean status of Romani-associated surnames. The figure plots the implied mean status of the *..y* surname group (squares), the top 20 most frequent names group (triangles), and the Romani-associated group (Xs). The vertical axis represents standard deviation units of social status. The colors and the sizes of the symbols represent data sources (large, maroon: doctors; medium, dark blue: technical PhD; small, light blue: technical masters

We separately look at the surnames associated with the Romani minority, and identified by their substantial increase in frequency from 1998 to 2016, and find an unexpected absence of regression to the mean. In Fig. 7 we plot the estimated status of the Romani-associated names (marked by an X) among medical doctors and technical university graduates, contrasted with the same figures for the *..y* ending surnames (marked by squares) and the top 20 most frequent surnames (marked by triangles) among the same elite groups. While the previously studied high and low-status surname groups both converge to the social mean over time, the Romani-associated surname group, which was already below-average status in the 1950s, actually diverges from the mean over time, implying a “convergence” rate *ρ* above unity. This is a truly striking result.

Not only does this indicate that the Romani minority experienced an ever-declining social status in the study period, but it also implies that not accounting for this fact will result in an overestimation of social mobility for the rest of the low-status groups. Though census-grade statistics are not available on the subject (Hungarian law strictly forbids ethnic profiling), estimates of the size of the Romani community indicate that it is a rapidly growing part of Hungarian society, currently comprising about 9% of all Hungarians.^29^ If a group that is increasing in size is also consistently diverging from the mean downwards, that means that other low-status groups that do converge towards the mean effectively do not “compete” against an increasing chunk of society. Not accounting for this would lead to an overestimation of social mobility among low-status groups. We show these alternative sets of (biased) results in the Appendix of Bukowski et al. (2021, Section C.1, Figures A4 to A9 on pages 67–70).

### General elites

We now turn to two sets of elite names that are not directly connected to education. The first is the set of inventors’ names in the PATSTAT database. The second is the set of names that appeared in the “Who is Who” books as a proxy for “famous people” in general. Again, the baseline results are presented relative to the non-Romani population. To streamline the presentation of the results, we only present the status change figures analogous to Figs. 2–6.

Tables A7 and A8 in Bukowski et al. (2021, pages 78–79) show the descriptive tables analogous to Table 2 with the evolution of the relative representation of the two high-status and two low-status surname groups and their implied mean status over time. In both cases, we account for the change in the relative “eliteness” of the general elites by fixing them at 1% at the first decade where the data was available and then adjust them by the relative size of the subsequent cohorts. Alternative specifications of the figures (with eliteness fixed as 1% for the whole study period) can be found in Section C.2 in Bukowski et al. (2021, Figures A14 and A15 on pages 72–73).

**Fig. 8:**
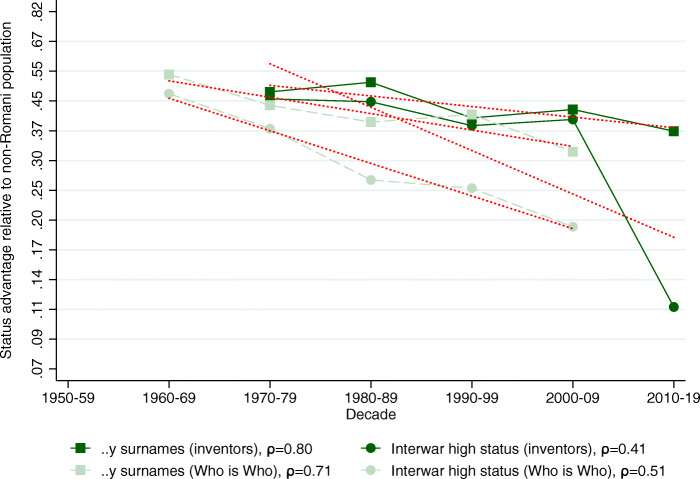
Status persistence of advantaged surnames among general elites. The figure plots the implied mean status advantage of the high-status names (in standard deviation units) and a linear fit. The data points can be found in Columns (1) and (2) of Tables A7 and A8 in Bukowski et al. (2021, pages 78–79). The status advantage is shown on a logarithmic scale on the vertical axis. The legends contain the calculated *ρ* intergenerational correlation coefficients of status by surname group. A lower correlation means less status persistence, which means more social mobility (steeper line in absolute value)

Figure 8 plots the decadal status estimates for the high-status groups in general elites. We see a very similar pattern to what we have seen with the educational elites. The estimated status persistence is higher for the *..y* surname group (.80 for inventors and .71 for Who is Who); lower and more noisily estimated with the interwar high-status groups: 0.51 for Who is Who and 0.41 for inventors. The abnormally low 0.41 coefficient is due to a singular outlier in the last decade created by interwar high-status names among inventors, otherwise, the group closely followed the pattern of the *..y* ending surname group, where persistence was twice as high.

**Fig. 9:**
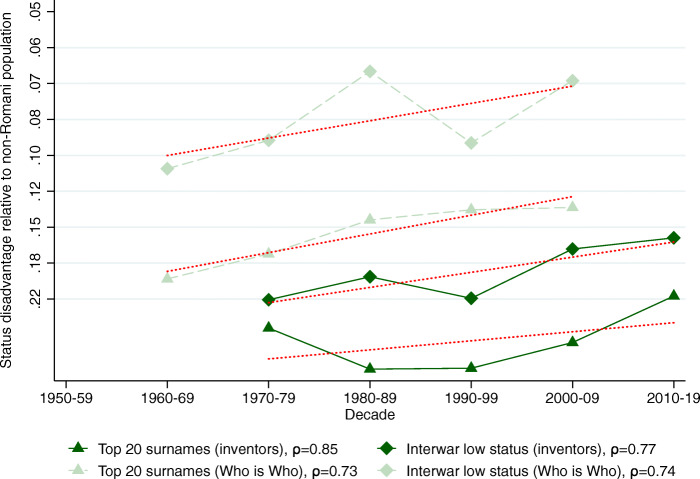
Status persistence of disadvantaged surnames among general elites. The figure plots the implied mean status advantage of the low-status names (in standard deviation units) and a linear fit. The data points can be found in Columns (1) and (2) of Tables A7 and A8 in Bukowski et al. (2021, pages 78–79). The status advantage is shown on a logarithmic scale on the vertical axis. The legends contain the calculated *ρ* intergenerational correlation coefficients of status by surname group. A lower correlation means less status persistence, which means more social mobility (steeper line in absolute value)

The low-status names in Fig. 9 also paint a remarkably consistent picture. The status persistence estimates are very high and also almost numerically identical for the top 20 most frequent surnames and the interwar low-status surname group both among inventors and in the Who is Who sample, ranging between 0.75 and 0.85. Another discernible common feature of this figure and the previous ones is an apparent lack of any visible trend break at the transition to capitalism.

### Where does transition to capitalism matter, and where does it not?

How did the regime change impact mobility? To answer this question, we contrast how relative representation on a yearly level changed around transition among political elites compared to medical doctors. Relative representation of surname groups among political elites is presented in Tables A9 (Hungarian Academy of Sciences) and A10 (Members of Parliament) in Bukowski et al. (2021, pages 80–81). We consider the year of the transition as 1990 for the political elites (the year of the first free and fair election), and 1996 as the year of transition for medical doctors (when the first cohort who started their studies after transition graduates).

**Fig. 10:**
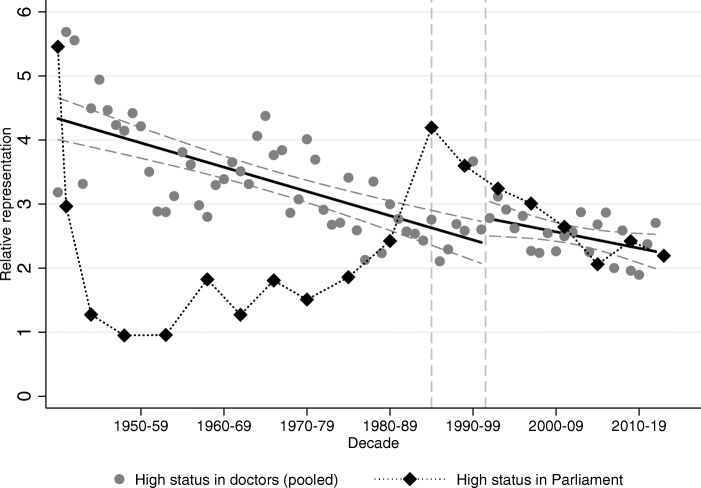
High-status names in Parliament vs. high-status names among doctors. The figure plots the relative representation of high-status names (both groups combined) among medical doctors (gray circles) and Members of Parliament (black diamonds). The vertical lines correspond to the regime change in 1990 and to the first year when a medical student who started school after the regime change would have graduated (1996)

**Fig. 11:**
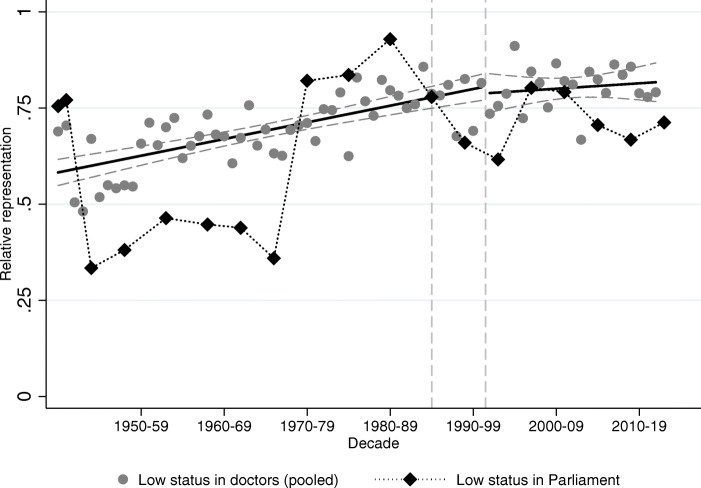
Low-status names in Parliament vs. low-status names among doctors. The figure plots relative representation of low-status names (both groups combined) among medical doctors (gray circles) and Members of Parliament (black diamonds). The vertical lines correspond to the regime change in 1990 and to the first year when a medical student who started school after the regime change would have graduated (1996)

Figures 10 and 11 show the relative representation of high and low-status surnames (respectively) among medical graduates (in gray) and Members of Parliament (in black). For this exercise, we pool all high-status names and all low-status names together to maximize statistical power. The relative representation equals 1 if the share of the name group is the same in Parliament (or among doctors) as it is in society; higher than 1 if the name is over-represented in Parliament (or among doctors), below 1 if under-represented. We connect black dots among election observations to represent the fact that there is a degree of continuity between Members of Parliament over time, while each gray dot represents a different cohort of medical graduates.

Figure 10 has two striking features. First, there is no break or level shift in the trend around which high-status names regress to the social mean among medical graduates. To highlight this, we draw 95-percent confidence bands around the trend estimated for the communist period and the trend estimated for the capitalist period. Second, while representation among doctors does not follow changing social and political regimes, the representation among the political elite does. The high-status names were still over-represented in Parliament in the first relatively free elections in 1945, while they were pushed to proportional representation under high Stalinism (the elections of 1949, 1953, and 1958, the first election after the Red Army suppressed the revolution in 1956). Oddly enough, as soon as the regime begins to thaw (from the 1960s), the share of high-status names starts to gradually increase to reach the same level of representation as among the doctors by 1985. During the first free and fair election their share jumps and starts gradually regressing to the trend represented by medical graduates.

Figure 11 plots the relative representations of the low-status names over time. Again, the first feature to note is the apparent lack of any effect of transition on social mobility as seen in relative representation among medical graduates. In Table A11 of Bukowski et al. (2021, page 82) we show that indeed there is no significant change in the level or the slope of the trend in relative representation around the regime change neither among high-status names nor among low-status names. The second interesting feature is again the course of the political representation of low-status surnames. These had similar representation in the social and political elites in the short-lived democratic period after World War II (elections of 1945 and 1947), then their political representation shrank below their social representation for the next twenty years in a political regime that was supposedly working to promote their social status. We do not have a full explanation for this, though we conjecture that the peasantry was heavily represented among the low-status surnames, and the attitude of Communists towards this group ranged from suspicious to overtly hostile. This changes during the late 70s, and from then on social and political representation of the low-status names remains very close to one another. It is also interesting that the representation of the low-status names in Parliament also fell below their representation among medical names during the Orbán-regime (from 2010 onward).

**Fig. 12:**
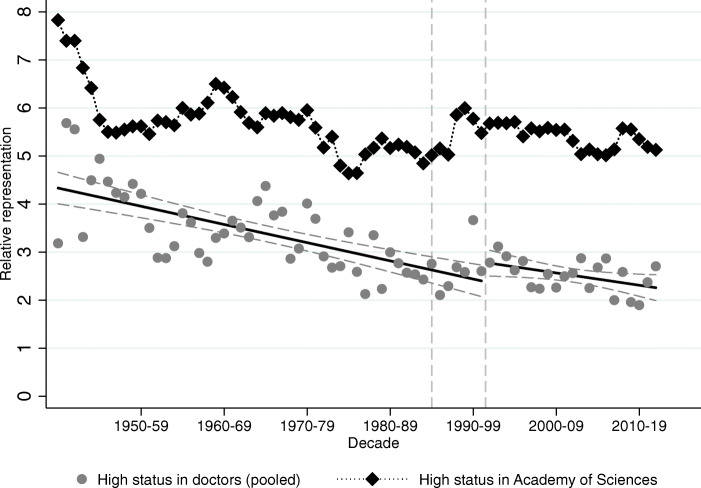
High-status names in the Academy of Sciences vs. high-status names among doctors. The figure plots relative representation of high-status names (both groups combined) among medical doctors (gray circles) and members of the Hungarian Academy of Sciences (black diamonds). The vertical lines correspond to the regime change in 1990 and to the first year when a medical student who started school after the regime change would have graduated (1996)

**Fig. 13:**
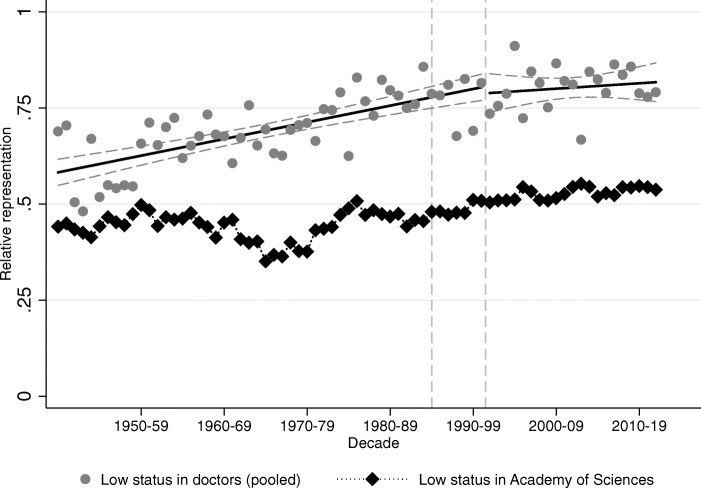
Low-status names in the Academy of Sciences vs. low-status names among doctors. The figure plots relative representation of low-status names (both groups combined) among medical doctors (gray circles) and members of the Hungarian Academy of Sciences (black diamonds). The vertical lines correspond to the regime change in 1990, and to the first year when a medical student who started school after the regime change would have graduated (1996)

We now turn to Figs. 12 and 13, where we plot the representation of the high and low-status names in the Hungarian Academy of Sciences against the backdrop of their representation among medical graduates. In Fig. 12 we see the same general pattern as in Fig. 10, namely, that regime changes cause changes in the representation of high-status names in the Academy, though the effect is more muted. An important difference is that high-status names are much more over-represented in science than they are in politics, and this does not even change during the worst years of Stalinist dictatorship. This is true even though communists expelled some members in 1949 because of their political sympathies.

Figure 13 confirms that indeed the Academy of Sciences is on average more elite than the Hungarian National Assembly, as the under-representation of low-status names is much striking here than it was either among Members of Parliament or medical doctors. However, the relative representation of the low-status names mostly evolved parallel to their representation among medical doctors, and we do not see any trend break at the regime changes of the twentieth century.

It is not possible to carry out such a formal test as above for any direct effect of the formal communist takeover in 1949 on the educational outcomes for the upper and lower surname groups. In part this is because it is much less clear which particular year one should use as *the* year of the regime change. It is certain that in the year 1949 the process was already complete, but Communists had been assuming effective control of the government and of important state institutions from 1946 on. So the break is actually the era from 1946 to 1949. Because of this, we have to anchor our estimates in the 1950s, by which time the harshest Stalinist policies were in place, so our estimates possibly miss some of the downward mobility in this era. On the other hand, this period was preceded by the significant disruptions of World War II, where medical school graduations were limited, and where the population shares of different groups were changing significantly.

Our measures of mobility within the communist regime take 1950–59 as the basis for measuring status, which might mean that our estimates miss some of the downward social mobility generated by the onset of communism. Since we are measuring people at age 25 graduating from medical school in the 1950s, these people would need to have graduated from high school sometime in the period 1943–1952. This cohort arguably still reflects the structure of pre-communist society. However, if the Stalinist regime in power between 1949 and 1956 had pursued explicit policies that barred from universities those of “bourgeois” social background (which we anecdotally know was the case), then we would have missed some of the social mobility created by the communist era.

However, if we refer to Table 2 above, and look just at the most robustly measured high- and low-status groups, the *..y* and top 20 surname groups, we see that there is surprisingly little change in the relative representation of these surnames among medical graduates between the 1940s and 1950s. There is no sign that under the communist regime in the 1950s the share of *..y* surnames among medical graduates declined unusually. Nor is there a sign of any unusual influx of the sons and daughters of the proletariat bearing the common surnames of Hungary. For the medical schools, communism looks like business as usual in terms of social mobility - a gradual replacement of the children of traditionally elite groups by the children of the traditional lower classes.

## Conclusions

At the end of WWII, and the formal emergence of a communist regime in 1949, Hungary had a social class structure that could trace its origins to at least the early nineteenth century. The descendants of the traditional aristocracy were still heavily over-represented in the educational elites, and the lower classes of the nineteenth century were still underrepresented in these same educational elites.

What happened to these upper-class and underclass groups, as indicated by their surnames, in the two very different ideological regimes of postwar Hungary: communism (during 1949–1989) and free market capitalism (during 1989–2017)? We show using surnames that there was very slow mobility within the non-Romani population in Hungary across both these regimes, with an intergenerational correlation in educational status that was in the range 0.6–0.8. The result was that even by 2010–17 someone with a surname inherited from the eighteenth century upper class was still 2.5 times more likely to gain a medical qualification than the average non-Romani person. And someone with a common Hungarian surname was 20% less likely to gain a medical qualification than the average of the non-Romani population.

Our findings show that, in the case of educational elites, social mobility rates under communism were the same as in the subsequent capitalist regime. These results seem to be at ad odds with our application of the Becker and Tomes model to regime changes. We must acknowledge that the economic models of social mobility focus on intergenerational correlation of income, while our measurement of social mobility is based on social status. While there is a clear positive correlation between our conceptualizations of elite social status (e.g. doctors, inventors, politicians) and income in each regime, it could be the case that changes in the relative earnings of occupations across the social regimes might blur the comparison. For instance, if doctors were relatively underpaid (compared to other professions) during communism than in capitalism, then the high persistence of social status of certain groups measured by the share among doctors in this period might not go in hand with the persistence of status as measured by income. However, this is not what the literature suggests, in socialist Yugoslavia, for instance, white-collar high-skill professions were at the top of the income distribution (Novokmet 2017).

Our results are more in line with the literature in sociology that argues that differences in the access to human capital and cultural capital reproduce pre-communist era inequalities over the long run (Böröcz and Southworth 1996), and these are passed on very similarly in all industrialized countries regardless of the social regime (Treiman and Yip 1989). There is also a long history of thought arguing that although communists declared that the working class ruled in their regime, in reality, it was increasingly dominated by the intelligentsia (Konrád and Szelényi 1979). Böröcz and Southworth (1996) note that this “takeover” happened exactly during the time when the state cut back on its education budget in the 1970s (Andorka and Harcsa 1990).

Finally, it is important to highlight what our paper does not say. We do not make any claim that “communism had no effect” on social stratification in Hungary, which would obviously be untrue. Our findings rather show that even such an extremely high cost-high effort “reform” (involving, among other “policies”, the confiscation of virtually all private property, abolishing free elections, and physical persecution of previous elites) aimed to fundamentally transform society could not completely eliminate pre-existing social differences, which were reproduced over subsequent generations. This is in line with the findings of Alesina et al. (2020), who come to similar conclusions looking at the communist experiment in China using a different methodology.

Consequently, our findings have implications for the debate on the future of capitalism and policies aimed to increase economic opportunities. They throw into doubt the assumption that institutional changes will fundamentally change rates of social mobility. Interestingly, the same is not true for income inequality, which fell significantly after the introduction of socialist systems in Hungary and other Eastern European countries (Mavridis and Mosberger 2017; Bukowski and Novokmet 2021; Novokmet et al. 2018). This suggests that the relationship between inequality and social mobility might be more complex than the “Great Gatsby” curve suggests (Krueger 2012), and that privileged groups might be able to protect their status even after losing some of their economic advantage.

## Footnotes

1 Recent studies looking at within-country cross-regional determinants of social mobility point to a positive effect of social capital, education and economic activity, and a negative effect of inequality (Chetty et al. 2014; Chetty et al. 2014; Corak 2013; Güell et al. 2018).

2 Individual-level estimates usually show a correlation around 0.2–0.3 across generations (Corak 2013), while group-level estimates are in the 0.7–0.8 range (Clark 2015), implying stronger persistence of status. This is not a contradiction, as individual- and group-level social mobility are related but distinct concepts (Solon 2018). See Section 4.1 for more detail on this, and Mazumder (2005) for a discussion on how measurement error might affect the individual-level estimates. Finally, in Appendix B of the working paper version of this article (page 65 in Bukowski et al.^2021^) we show benchmark estimates of Hungarian social mobility from earlier sociological papers.

3 Low rates of social mobility during communism are also reported for China by Hao and Clark (2012). The authors interpret it as the importance of kin networks in the intergenerational transmission of status.

4 That is, when *α*(1 + *λ*) < 1.

5 Gerber and Hout (2004) find that workers who experienced upward mobility during the Soviet era regressed to lower occupational status after transition; Betthäuser (2019) shows that East German educational inequality quickly caught up to higher West German levels upon re-unification.

6 Figures A10 to A15 on pages 70 to 73 in Bukowski et al. (2021).

7 Define the relative representation of group g as $RR_{g}=\frac {\#g in elite}{\#elite}/\frac {\#g}{\#pop}$. The relative representation is informative in itself, as it shows how over-represented (or under-represented) is group g in the elite compared to its population share. Theoretically we can think of the relative representation as follows: $RR_{g}=\frac {P(group=g|elite)}{P(group=g)}$. We can calculate the share of population in certain elite $P(elite)=\frac {\#elite}{\#pop}$ (or assume that they represent a top portion of the population). Multiplying the relative representation with *P*(*e**l**i**t**e*) we obtain:

$$ \frac{P(group=g|elite)P(elite)}{P(group=g)}=\frac{P(group=g\cap elite)}{P(group=g)}=P(elite|group=g)=P_{elite}^{g} $$

8 In particular, we carry out robustness checks where we set the educational status threshold for doctors at 0.5% and 2%, and where we allow the upper- and underclass surname groups to have a variance of status that is 0.8 or 1.2 times that of the population as a whole.

9 With the exception of names ending in *..gy*, *..ly*, *..ny* and *..ty*, which are not *..y* ending names, as these letter pairs form single consonants in the Hungarian language.

10 The 1720 census is available at https://adatbazisokonline.hu/adatbazis/az-1720_-evi-orszagos-osszeiras; we digitalized the 1755 census of the nobility from Illésy (1902); the list of the land owners in 1767 is from the Urbarium of 1767, available at https://archives.hungaricana.hu/en/urberi/.

11 See Chapter 3 of Gáspár (2019) for a more detailed description.

12 As was the family of the Nobel-laurate György Hevesy. Another, similar avenue of getting a *..y* name was the admission to the Knightly Order of the Vitéz during the Horthy regime. As the head of state was legally just regent of the Kingdom of Hungary, he had no constitutional authority to make noblemen. The Order of the Vitéz filled the same purpose.

13 We have access to a list of high school graduates compiled by historians Zsuzsa Bíró, Viktor Karády, and Péter Tibor Nagy. The data set is the one presented in Karády (2012). We owe gratitude to Viktor Karády and Péter Tibor Nagy for allowing us to use their data on high school graduates. We estimate population shares of names from a large sample of wedding records between 1895 and 1939. We are grateful to the Hungarian Association for Family History Research for giving us access to this data set.

14 As we consider any surname that was in the top 20 in any of our twentieth century data points, the list actually has 23 surnames.

15 Professions: Juhász — shepherd, Kovács — smith, Lakatos - locksmith, Mészáros — butcher, Molnár — miller, Szabó — taylor, Takács- weaver, Varga — leatherworker; Physical characteristics: Farkas — wolf (hunter, or has the physical properties of a wolf), Fehér — white, Fekete — black, Kis — little, Nagy — big, Balogh — left-handed, unlucky Ethnicity or culture: Tót — Slovak, generic term for anyone of Slavic origin in the middle ages, Német — German, Oláh — Romanian, Rácz — Serbian or anyone from the south, Török — Turkish The rest: Pap — priest (serf belonging to a church property), Simon — Biblical origin, Szilágyi — “from Szilágy”; Szilágy is a common settlement name and later the name of a county. Source: Hajdú (2010)

16 The average growth rate identified as Romani by the Encyclopedia of Surnames between 1998 and 2016 was 17%. The names that had the highest growth rates between 1998 and 2016 were those potentially attributable to recent immigration (e.g. Asian and Middle Eastern names). To avoid confounding these with Romani names, we also imposed a requirement that a name included in our set had to have at least as many occurrences in 1998 as the least common name that was associated with the Romani in the Encyclopedia.

17 The records were digitized by the Hungarian Society for Family History Research, who kindly shared the yearly surname distribution. The dataset is at http://www.macse.hu/databases/en/default.aspx.

18 Physician are trained at four different Hungarian universities. See Kapronczay (2013) and Kiss (2018) and Péterffy (2016) for a brief history.

19 Source:https://www.ksh.hu/interaktiv/korfak/orszag.html

20 The yearbooks are available at https://library.hungaricana.hu/hu/collection/egyetemi_jegyzokonyvek_bme_evkonyv/

21 Usually patenting an invention is connected with several applications, called a *family*. We collapse the data to families instead of individual patents.

22 We thank Miklós Koren, Ádám Szeidl and András Vereckei for letting us use these data.

23 All of these are available online as PDF-s at the Hungaricana Hungarian Cultural Heritage Portal: Almanacs: https://library.hungaricana.hu/hu/collection/ogyk_almanach/ Address books: https://library.hungaricana.hu/hu/collection/ogyk_lakaskonyv/ Session records: https://library.hungaricana.hu/hu/collection/orszaggyulesi_dokumentumok/

24 https://www.parlament.hu/web/guest/kepviselok-elozo-ciklusbeli-adatai

25 Available from Markó et al. (2003); more recent appointments can be found on the homepage, see https://mta.hu/mta_tagjai.

26 The general elite data includes only Hungarian nationals, we did not have to adjust it. Members of Parliament are also Hungarian nationals. While the Hungarian Academy of Sciences confers honorary memberships to scholars in other countries, we limit our attention to full and corresponding members, who are all Hungarian nationals as well.

27 The Hungarian Jewry was among those Jewish communities of Europe that suffered the highest loss of life both in absolute and relative terms during the Holocaust (Braham 1981). After the war, hundreds of thousands of ethnic Germans fled or were driven out of Hungary. At the same time, neighboring countries, such as Czechoslovakia, expelled large chunks of the ethnic Hungarian population. In the meantime, many people from the Slavic minorities in Hungary decided to emigrate to neighboring Slavic-majority countries. Many of those who had Jewish, German or Slavic backgrounds and decided to remain chose to adopt a Hungarian surname. This movement lost intensity by the 1950s (Karády and Kozma 2002)

28 The tables show the raw data; the adjusted data is shown in Table A4 of Bukowski et al. (2021) (page 75).

29 The few existing and reliable estimates tell us that in 1893 the Romani population share within modern borders of Hungary was 1.1%. By 1978 this had risen to 3%. But by 1993 this was 4.4%, and by 2012 8.8%. See Kertesi and Kézdi (1998), Kocsis and Kovács (1999) and Pénzes et al. (2018)
